# “Cost” of virginity in wild *Drosophila melanogaster* females

**DOI:** 10.1002/ece3.54

**Published:** 2011-12

**Authors:** Therese Ann Markow

**Affiliations:** Division of Biological Sciences, University of CaliforniaLa Jolla, San Diego, California

**Keywords:** Costs, *drosophila*, field, longevity, mating

## Abstract

Laboratory studies have revealed a significant “cost of mating” to *Drosophila melanogaster* females in the form of reduced longevity. The effect is attributable to nonsperm components of the ejaculate. Female *D. melanogaster* are known to mate up to six times in nature, and given that they do not typically remate daily, it raises the question as to the extent to which the longevity of wild mated females is reduced. Here I addressed this question by comparing the longevity of wild virgin females, collected as they emerged from rotting fruit, to the longevity of randomly collected mature females at the same site. Because the randomly collected females all were inseminated and were fully pigmented at the time of collection, they already were older than the virgins when the experiment began. Contrary to expectations from laboratory studies, the older, mated females lived significantly longer than the virgins. Rather than a “cost of mating,” there appears to be a “cost of virginity” to female *D. melanogaster* in the wild.

## Introduction

In the laboratory, reduction in life span of female *Drosophila melanogaster* is associated with mating (Fowler and Partridge 1989; Rice 1996; Pitnick and Garcia–Gonzalez 2002; Markow and O'Grady 2005). Mated females in these studies die, on average, 4–8 days earlier than control females. Components of the male ejaculate, in particular the sex peptide, a male accessory gland product, appear responsible for this “cost of mating” to females (Chapman et al. 1995; Wigby and Chapman 2005), as their ablation through genetic tools eliminates the effect.

At the same time, in nature female *D. melanogaster* remate when their sperm load has become reduced (Gromko and Markow 1993) and the majority of females in nature remate at least two to six times in their lifetimes (Milkman and Zeitler 1974; Stalker 1976; Griffiths et al. 1982; Marks et al. 1988; Harshman and Clark 1998, Imhof et al. 1998). The average number of eggs female *D. melanogaster* lay per day can reach two times the number of ovarioles (King 1970; Cohet and David 1978). Given that the average number of ovarioles in female *D. melanogaster* is between 35 and 40 (Robertson 1957; King 1970), inseminated females lay approximately 80 eggs a day. Of the 4000 sperms *D. melanogaster* females receive upon copulating, they store 400–500 of these and the efficiency of the use of the stored sperm is about 50% (Gilbert et al. 1981). Thus, the reduction in stored sperm and remating in nature would be expected to occur every 2–5 days provided females encounter suitable oviposition sites. Thus, having remated three or four times, as evidenced by molecular studies of paternity (Imhof et al. 1998), females collected at random in nature would have received one or more doses of “life-shortening” seminal substances as well as sperm and would on average be several days older than newly emerging virgin females. In addition, life in nature presents flies with additional challenges to survival, such as temperature and humidity fluctuations, parasites, and unpredictable food sources compared to the constancy of the laboratory.

These observations from the laboratory and the field seem inconsistent. If mating reduces female life span, how do mated females in nature live sufficiently long to remate multiple times? At present, no data exist regarding how long females collected from nature live, but in the laboratory, the life-shortening effects of mating are observed within a few days (Fowler and Partridge 1989; Chapman et al. 1995; Wigby and Chapman 2005). If mating reduces life span, mated females collected in the field should not live as long as virgins from the same population.Table 1

**Table 1:** Mean day at death for virgin and randomly collected adult female *Drosophila melanogaster* and the mean number of progeny produced by mated females

Female type	Replication	Mean ± SE (number of females) day at death	Mean progeny number ± SE (*n*)
Virgin	1	48.34 ± 1.14 (19)	
Random adult	1	54.74 ± 1.34 (26)	162.4 ± 12.6 (26)
		*F* = 13.06, *P* < 0.0008	
Virgin	2	46.7 ± 1.36 (21)	
Random	2	53.4 ± 0.93 (30)	156.2 ± 11.05 (30)
		*F* = 17.82, *P* < 0.0001	

To test this prediction, I collected emerging virgin female and mature female *D. melanogaster* feeding at random from the same rotting fruit pile and recorded how long each female lived as well as how many progeny she produced. Although it is not possible to reliably assign exact ages to wild caught *Drosophila*, mature females must be older, to variable extents, than newly emerged teneral females. In every case, because randomly collected mature females produced viable progeny, it was clear that they were not virgins, allowing effect of mating on longevity of *D. melanogaster* females from natural populations of flies to be tested. In both replications of this experiment, virgin females died significantly sooner than did the mated females, the opposite of what is predicted from laboratory observations.

## Materials and Methods

### Study site

I sampled individuals at a residential yard in the town of Alamos, Sonora, Mexico, where a continuous population of *D. melanogaster* occurred at a rotting fruit pile to which I added the rinds of two cantaloupe melons, one honeydew melon, and the peelings and core of one pineapple every 3 days.

### Collection and handling of females

I performed two replications of this study. The first experiment began on January 18, 2011 and the second on February 28, 2011. Each began when I collected virgin (light-colored, emerging) and mature females on a single morning, all during a 45-min period. To collect individuals, I inspected the fruit pile for emerging virgins, gently aspirating them into individual unyeasted vials containing banana medium. At the same time, I carefully collected mature, fully pigmented female *D. melanogaster* as they fed upon and walked around on the fruit pile and aspirated them into individual unyeasted food vials. Each day I transferred the individual females to new unyeasted food vials and scored those who died. I counted the number of progeny each female produced.

### Statistical analysis

I performed all statistical analyses using JMP software Version 8.0 (SAS Institute, Carey, NC). Kaplan–Meier survival curves compared mean day of death of mature and virgin females using both Log Rank and Wilcoxon tests (a priori significance set at alpha < 0.05, ANOVA tested the relationship between day of death and productivity in the mature females).

## Results and Discussion

**Figure 1:**
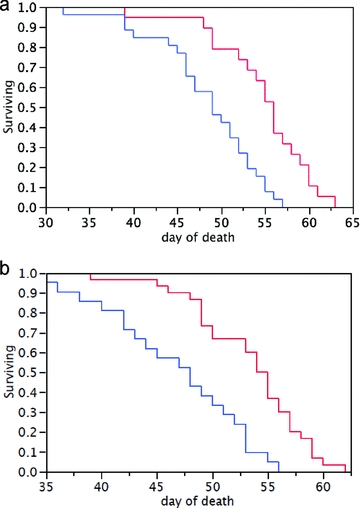
(A, B) Female survival after collection in the field for Replications 1 (top) and 2 (bottom). Virgin females are indicated by the blue line, randomly collected mated females are indicated by red. For Replication 1, Log-Rank χ^2^ = 15.16, df = 1, *P* < 0.0001; Wilcoxon χ^2^ = 12.22, df = 1, *P* < 0.0005, and for Replication 2, Log-Rank χ^2^ = 17.35, df = 1, *P* < 0.0001) and Wilcoxon χ^2^ = 16.03, df = 1, *P* < 0.0001.

All randomly collected mature females from both replications produced progeny indicating that these females were inseminated. No recently eclosed females produced progeny indicating that they were still virgins on capture. In contrast to what was expected, mated females lived significantly longer than the virgins in both replications of the experiment (Table 1). In the first replication, there were 19 virgin and 26 mated females and the mated females lived an average of approximately 6 days longer. In the second replication of 21 virgin and 30 mated females, the difference in mean day of death was similar, although both virgin and mated flies died an average of 1 day earlier than in the first trial (Table 1). Kaplan–Meier survival curves for the two replications are in Figure 1A and 1B, respectively. By both tests, Log-Rank (χ^2^ = 15.16, df = 1, *P* < 0.0001) and the Wilcoxon (χ^2^ = 12.22, df = 1, *P* < 0.0005), the difference in survival in the first replication was highly significant, with mated females living longer. In the second replication, both the Log-Rank (χ^2^ = 17.35, df = 1, *P* < 0.0001) and Wilcoxon (χ^2^ = 16.03, df = 1, *P* < 0.0001) tests also revealed that mated females lived significantly longer than virgins.

The range of progeny mature females produced in both replications was similar, from just a few eclosed offspring to over 250. Females producing only a few offspring may have been running out of sperm and thus possibly older than the others. If this was the case, they might be expected to die earlier. Among the mated females, however, longevity was not associated with the number of offspring a female produced (Replication 1, *R*^2^ = 0.0001, *F* = 0.002, *P* = 0.96; Replication 2, *R*^2^ = 0.09, *F* = 2.68, *P* = 0.11).

Clearly, the true age only of the virgin females could be known. Randomly collected females in nature, however, had to be older than the virgins, as they had mated and their cuticles were fully pigmented. According to all previous studies of paternity in wild *D. melanogaster* females (Milkman and Zeitler 1974; Stalker 1976; Griffiths et al. 1982; Marks et al. 1988; Harshman and Clark 1998, Imhof et al. 1998), the majority of randomly caught females have sperm from more than one male. Thus, it is reasonable to assume that (1) the randomly collected mature females were at least several days older than the virgins, (2) many had received at least double amounts of presumed “life-shortening” ejaculate substances, and thus (3) should have died significantly earlier relative to the virgins.

Why, then, did virgins die before mated females? It could be argued that the act of aspirating virgin females was somehow more stressful to them than to older females, resulting in their relatively earlier death. However, I used extreme care during collection, employing wide diameter aspirators, exerting hardly any suction. Aspirated females were allowed to crawl from the aspirators into the food vials, rather than being blown. An additional possibility is that in nature, the male ejaculate may not be harmful to females or could actually contribute, via some yet unknown mechanism, to female longevity. For example, mated female *D. melanogaster* are more resistant than virgins to starvation (Goenaga et al. 2011) and in *D. mojavensis*, mated females are more desiccation resistant (Knowles et al. 2005), suggesting that stressors in nature change the metabolic consequences of mating. In the present study, flies developed and mated in nature and the ecological histories and ages of the mated females are unknown. It is likely that at some point in their preadult and/or adult lives, their experiences and exposures were different than those of laboratory-reared and mated females. For example, female nutritional status is reported to influence the response to male ejaculate components (Fricke et al. 2010), and nutrition in nature is known to differ from standard laboratory diets (Markow et al. 1999; Jaenike and Markow 2003). Other environmental differences may also be involved. If, for example, immunity genes are already expressed in flies in nature, their induction by mating in the laboratory, and the suggested “cost” of this induction as the basis for reduced longevity (Innocenti and Morrow 2009), may not be relevant in nature. Furthermore, females in nature may receive less seminal fluid during a given copulation than those in laboratory experiments where virgin males are stored for several days prior to copulating. And finally, the genotypes of the flies used in laboratory studies are different. Some have carried mutant markers with potential and unknown pleiotropic effects on the reproductive process. The wild-type strains all have been in the laboratory for multiple generations, even though most investigators have been careful to maintain large out-crossed populations prior to the experiments. As Chapman et al. (2003) appropriately point out, however, the possibility of inbreeding can confound interpretations of these experiments. This might explain why the flies in both replications of the present study lived considerably longer than those in any of the laboratory studies of the “cost of mating” in *D. melanogaster.* A more likely contributor to the greater longevity of all flies in the present study relative to others is the fact that other studies supplemented the food vials of the ageing flies with grains of yeast while I did not. Caloric restriction is known to increase *Drosophila* longevity, especially restriction of yeast (Min et al. 2007). Additionally, long-term laboratory maintenance can lead to laboratory adaptations involving life-history characters (Partridge et al. 1995; Sgrò and Partridge 2001).

Regardless of the underlying explanation for the greater relative longevity of mated females, the observation calls into question assumptions underlying sexually antagonistic coevolution and arms races between the male ejaculate and the female's response to it. An assumed “cost of mating” for females in *D. melanogaster*, as observed in the laboratory, has been central to this literature (Rice et al. 2006; Innocenti and Morrow 2009; Edward et al. 2010) along with experiments demonstrating the evolution of female resistance (Wigby and Chapman 2007; Morrow et al. 2008). However, these “costs,” while clearly established in *D. melanogaster* in the laboratory, including in my own laboratory (Markow and O'Grady 2005), were not observed in laboratory matings in *D. pseudoobscura.*Gowaty et al. (2010) found no difference in female longevity between *D. pseudoobscura* females mated once or multiple times. Because *D. melanogaster* females do not experience a decrement in the longevity component of fitness in nature, and females of other *Drosophila* species do not experience reduced longevity from mating in the laboratory, perhaps it is appropriate to reexamine the evolutionary and ecological contexts of female mating costs and the sexually antagonistic processes assumed to ensue.
